# The Generic Inhibitory Function of Corollary Discharge in Motor Intention: Evidence from the Modulation Effects of Speech Preparation on the Late Components of Auditory Neural Responses

**DOI:** 10.1523/ENEURO.0309-22.2022

**Published:** 2022-12-07

**Authors:** Xiaodan Zheng, Hao Zhu, Siqi Li, Xing Tian

**Affiliations:** 1 Shanghai Key Laboratory of Brain Functional Genomics (Ministry of Education), School of Psychology and Cognitive Science, East China Normal University, Shanghai 200062, People’s Republic of China; 2 NYU-ECNU Institute of Brain and Cognitive Science, New York University Shanghai, Shanghai 200062, People’s Republic of China; 3 Division of Arts and Sciences, New York University Shanghai, Shanghai 200122, People’s Republic of China

**Keywords:** action-induced sensory suppression, agency, internal forward model, motor control, sensorimotor integration

## Abstract

The importance of action–perception loops necessitates efficient computations linking motor and sensory systems. Corollary discharge (CD), a concept in motor-to-sensory transformation, has been proposed to predict the sensory consequences of actions for efficient motor and cognitive control. The predictive computation has been assumed to realize via inhibiting sensory reafference when actions are executed. Continuous control throughout the course of action demands inhibitory function ubiquitously on all potential reafference when sensory consequences are not available before execution. However, the temporal and functional characteristics of CD are unclear. When does CD begin to operate? To what extent does CD inhibit sensory processes? How is the inhibitory function implemented in neural computation? Using a delayed articulation paradigm with three types of auditory probes (speech, nonspeech, and nonhuman sounds) in an electroencephalography experiment with 20 human participants (7 males), we found that preparing to speak without knowing what to say (general preparation) suppressed neural responses to each type of auditory probe, suggesting a generic inhibitory function of CD in motor intention. Moreover, power and phase coherence in low-frequency bands (1–8 Hz) were both suppressed, indicating that inhibition was mediated by dampening response amplitude and adding temporal variance to sensory processes. Furthermore, inhibition was stronger for sounds that humans can produce than nonhuman sounds, hinting that the generic inhibitory function of CD is regulated by the established motor–sensory associations. These results suggest a functional and temporal granularity of corollary discharge that mediates multifaceted computations in motor and cognitive control.

## Significance Statement

The feeling and actual control of one’s body are linked to the same phenomenon of sensorimotor interaction—sensory processes of self-induced stimuli are attenuated by a copy of motor signals, coined as corollary discharge (CD). However, when, to what extent, and how CD inhibits sensory processes remain unclear. Using a delayed articulation paradigm in an EEG experiment, we found that CD inhibited all speech, nonspeech, and nonhuman sounds even when participants intended to speak, with stronger inhibition of the sounds that humans can produce. The inhibition was mediated by dampening response amplitude and adding temporal variance in low-frequency neural responses to sensory stimuli. These results suggest functional granularity of CD throughout the course of actions for motor control.

## Introduction

The efficient interplay of action and perception is an adaptive trait in any organism for survival. The importance manifests through evolution and engraves dedicated neural computational pathways linking motor and sensory systems (Crapse and Sommer, 2008). One of such functional computations has been theorized as the internal forward model (von Helmholtz, 1910; Wolpert and Ghahramani, 2000)—a copy of motor signals, coined as “corollary discharge” (CD; Sperry, 1950) or “efference copy” (EC; von Holst and Mittelstaedt, 1950), transmits to sensory systems to predict the sensory consequences of actions (Kawato, 1999; Schubotz, 2007). Such predictive functions of the internal forward model have been implied as canonical computations mediating visual perception (Ross et al., 2001; Sommer and Wurtz, 2006), motor control (Wolpert and Miall, 1996), speech production (Guenther, 1995; Houde and Nagarajan, 2011; Hickok, 2012), and higher-order cognitive functions such as mental imagery and agency (Desmurget et al., 2009; Tian and Poeppel, 2010; Kilteni et al., 2018).

**Figure 1.**
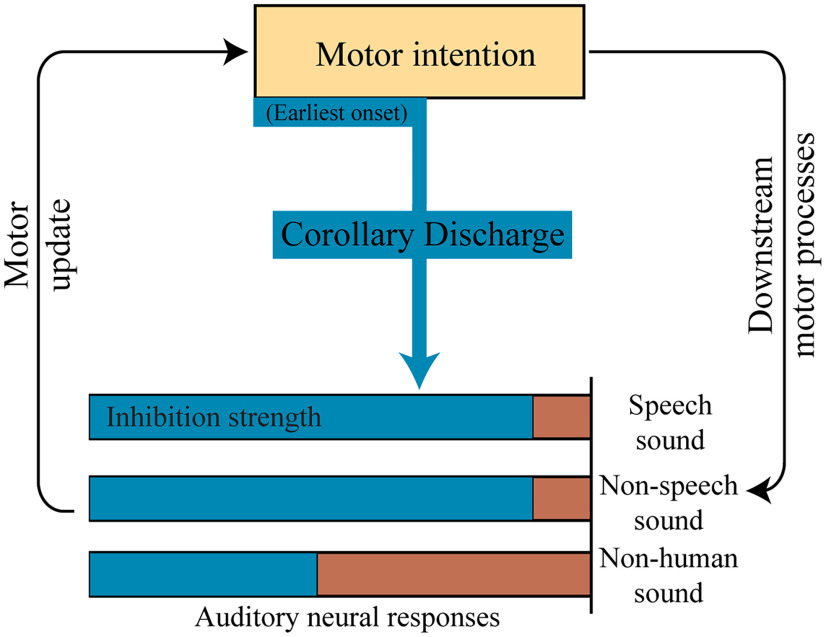
A schematic of the hypothesis about the generic inhibitory functions of CD. CD can be available as early as in the motor intention stage and throughout the entire course of action. CD inhibits sensory processing (demonstrated in blue). The inhibition function of CD could be nonspecific (generic) to all sounds at the beginning of the action course (motor intention) and becomes stronger and specific to the sensory consequences of actions. The generic inhibition function of CD may also depend on the established associations between actions and their sensory consequences—the strength of generic inhibition on the sensory process may depend on feature overlaps between feedback stimuli and the sensory representation that can be induced by actions performed by an agent. Specific in the auditory domain, the generic inhibition function of CD may suppress nonhuman sound less than for speech and nonspeech sounds that humans can produce, as indicated by the shorter blue bar in nonhuman sound.

The operation of the internal forward model has been assumed to rely on the inhibitory modulation of the CD and EC on sensory processing [Blakemore and Decety, 2001; Houde et al., 2002; Tian et al., 2018 (but also see exceptions of enhancement modulation in recent empirical and theoretical studies; Li et al., 2020; Press et al., 2022)]. Recently, an updated theoretical framework has been proposed by considering distinct modulatory functions of CD and EC throughout the time course of actions (Li et al., 2020). Specifically, EC is available after motor encoding and includes detailed action codes that selectively enhance the processing sensitivity of the sensory reafference. Whereas, CD exerts an inhibitory function and is available throughout the course of actions (Fig. 1). CD does not depend on specific information and is available as early as in motor intention to inhibit sensory consequences caused by all possible actions that an agent can perform—the generic inhibitory function of CD.

The early onset of CD has been supported by empirical results, but the generic inhibition is equivocal. In motor intention when participants prepared to speak but did not know what to say (general preparation), CD was generated in this earliest stage of actions and suppressed the neural responses to auditory syllables but not pure tones (Li et al., 2020). The mixed results could be because the CD induced in that study was not “general” enough—participants were only asked to pronounce syllables in subsequent articulation tasks, and hence the CD in general preparation could contain categorical information. Moreover, the generic inhibition of CD may be constrained by the distance between sensory feedback and the possible sensory consequences caused by the repertoire of actions that an agent can perform. Pure tones only partially overlap with features of the tones that humans articulate and hence could be less inhibited than sounds that humans normally produce (Fig. 1, shorter blue bar for nonhuman sound). A recent study found that the strength of suppression to auditory responses decreased as the frequency of tones deviated from the standard frequency of action consequence (Schneider et al., 2018). This evidence offers hints supporting our conjecture of the gradient suppression effects.

How CD exerts the inhibitory function is also unclear. Auditory processes can operate in temporal or rate codes (Lu et al., 2001). The modulation effects can be manifested by altering the magnitude or temporal aspects of responses (Grill-Spector et al., 2006). Specifically, the effects can be a result of direct gain modulation on response magnitude. Numerous studies have demonstrated that manual actions and speaking dampen the amplitude of neural responses to sounds (Houde et al., 2002; Baess et al., 2011). Whereas in the temporal dimension, it has been suggested that the phase of neural oscillations can be reset and aligned with upcoming external stimuli to boost the sensitivity of neural encoding (Schroeder and Lakatos, 2009; Giraud and Poeppel, 2012; Tomassini et al., 2017; Teng et al., 2020). If CD influences the alignment between neural phase and auditory stimuli, similar suppression effects can be achieved. Therefore, the generic inhibition of CD can potentially dampen response power or increase temporal variance in responses to sensory feedback.

To examine the hypothesis of generic inhibitory function and neural mechanisms of CD (Li et al., 2020), we adopted the delayed articulation paradigm and excluded categorical information from CD in general preparation by asking participants to produce three types of sounds in subsequent articulation task—a speech sound of syllable/ba/, a nonspeech sound of cough, and a humming tone that simulated a nonhuman sound of pure tone. According to the hypothesis of generic inhibition, general preparation would suppress the neural responses to all types of auditory probes, but less to pure tone. Moreover, the time–frequency analysis would reveal whether the inhibitory function was realized by dampening response amplitude or increasing temporal variance in sensory processes.

## Materials and Methods

### Participants

Twenty right-handed volunteers (7 males; age range, 19–25 years; mean age, 22.2 years) participated in the experiment. The sample size was determined as the same number of participants in the target comparison study that used similar paradigms (Li et al., 2020). All participants had normal hearing (self-reported). They received monetary compensation for their participation. Written informed consent was obtained from every participant before the experiment. This study was approved by the institutional review board at New York University Shanghai.

The sample size was predetermined to be 20 based on previous studies that investigated similar questions of action-induced suppression (Houde et al., 2002; Aliu et al., 2009; Horváth et al., 2012). Using G*power (Faul et al., 2007) to estimate the sample size based on the effect size (*d* = 0.8660) observed in the study by Houde et al. (2002), we found a sample size of 13, which was required to have 80% power at an α level of 0.05. Therefore, our sample size is large enough to replicate the action-induced suppression effect. We further calculated the statistical power of the present study using G*power to verify that we had enough power. We found that the present study had 94.84% power with a sample size of 20 at an α level of 0.05, based on the effect size (0.847) of the present EEG data.

### Materials

Three auditory tokens, each in every sound category—speech sound (a syllable/ba/), nonspeech sound (a cough sound), and nonhuman sound (500 Hz pure tone)—were used as auditory probes in the experiment. All stimuli were 400 ms in duration with a sampling frequency of 44.1k Hz, and their average (root mean square) intensity was normalized to 70 dB SPL using Praat. The auditory syllable (/ba/) was synthesized using the Neospeech web engine (www.neospeech.com) in a male voice, identical to the one used in the target comparison study (Li et al., 2020). The cough sound was recorded by a male native Mandarin speaker. The 500 Hz pure tone was generated using MATLAB. The frequency of the tone was selected by considering the usual lower bound of audiometry using pure tones as well as the range of the fundamental frequency of human vocal production. The pure tone was included so that we could investigate whether the modulation of CD on nonhuman sounds differs from human sounds.

### Procedures

We first summarize the procedure and its major differences from the target comparison study and then provide details next. The delayed-articulation paradigm was used in the experiment. Participants were required to make a general preparation—preparing to speak in the subsequent articulation task but not knowing what to say. We asked participants to produce three types of sounds in the articulation task (syllable, cough, and humming tone). In this case, the general preparation could be truly “general”—not constrained by a particular speech category but possibly extending to all sound categories that humans can produce, and hence our hypothesis about the generic inhibitory function of CD can be tested. The auditory probes that were presented during the preparation stage also included the three types of sounds to probe the modulation function of CD during general preparation.

**Figure 2.**
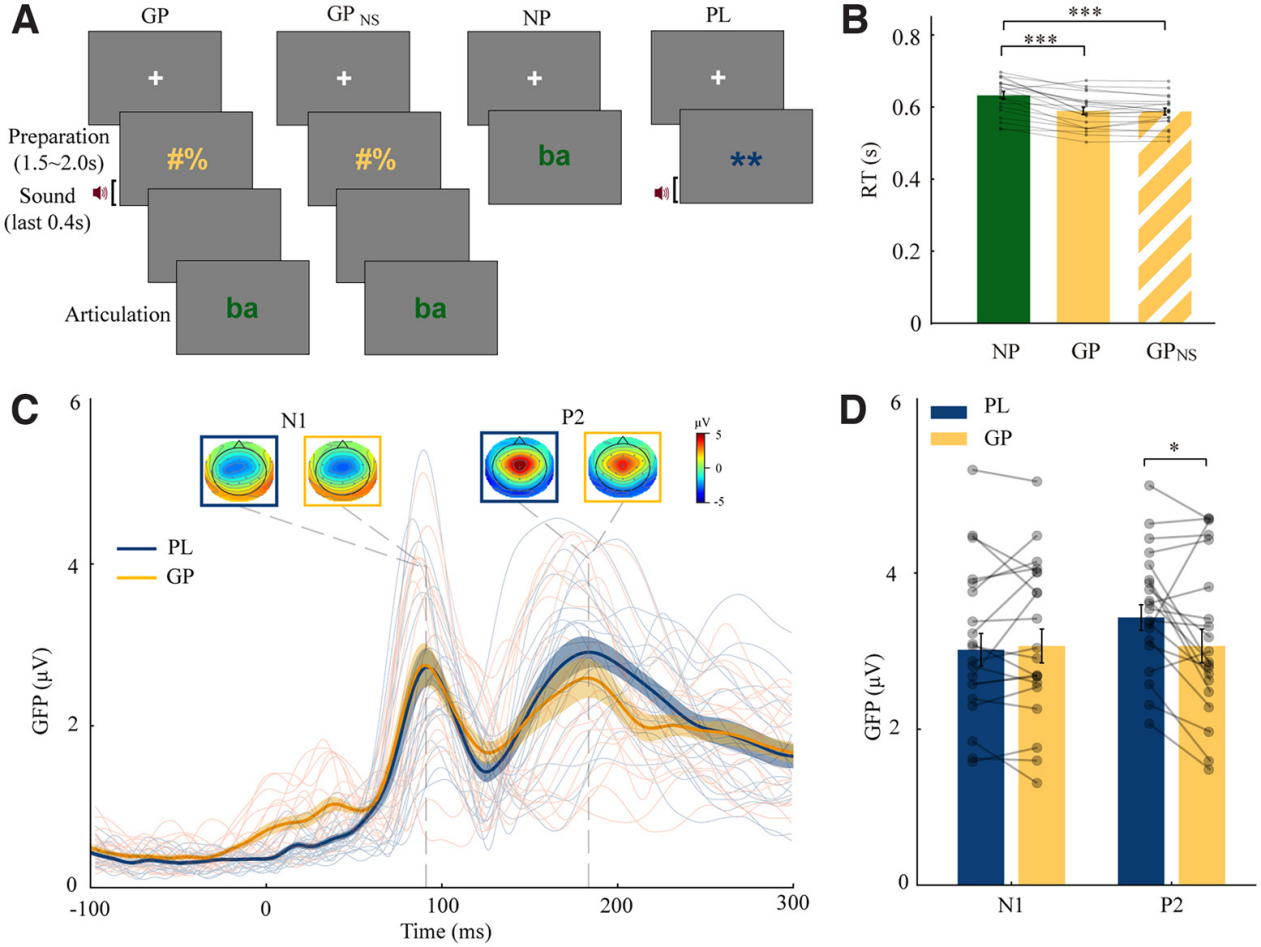
Experimental paradigms, and behavioral and ERP results. ***A***, Illustration of four types of trials. In GP trials, participants were asked to prepare to speak when two meaningless symbols were on screen. The symbols did not provide any information about what participants were going to say, and hence they generally prepare the action of speaking. An auditory probe (randomly selected from a syllable/ba/, a cough sound, and a 500 Hz pure tone) was presented at the end of the preparation stage to probe the modulatory effect of CD on auditory processes. When a green visual cue appeared, participants were asked to articulate accordingly. The visual cue “ba” is used as an example for illustration purposes; two other visual cues were included for producing cough and humming tone. GP_NS_ trials were identical to GP trials except that no auditory probe was presented during the preparation stage. In NP trials, participants performed the articulation task without preceding preparation. GP_NS_ and NP trials were used to control and quantify the general preparation. In the PL trials, participants were asked to passively listen to the auditory probes that were identical to those in GP trials. No preparation or articulation task was required in the PL trials. The PL trials were used to compare with auditory responses in GP trials to quantify the neural modulation effects of preparation (for details, see Materials and Methods) ***B***, Mean RTs across three conditions with individual data. Participants articulated faster in GP and GP_NS_ conditions than in NP condition, but no difference between GP and GP_NS_ conditions, suggesting that the performance of general preparation was independent of the auditory probes. Error bars indicate ±SEM. ***p < 0.001. ***C***, Grand average GFP waveforms and topographic responses for three types of auditory probes combined. Auditory N1 and P2 components were observed in each condition. Yellow and blue represent GP and PL conditions, respectively. Individual waveform responses are superimposed on the plot. ***D***, Mean N1 and P2 amplitudes in two conditions with individual data. The magnitude of the P2 component was significantly smaller in GP than that in PL, suggesting that general preparation suppressed the auditory responses. Error bars indicate ±SEM. *p < 0.05.

The detailed procedures are as follows. To examine the hypothesis and control confounding variables, four types of trials were included in the experiment: general preparation (GP) trials, GP with no sound (GP_NS_) trials, no preparation (NP) trials, and passive listening (PL) trials. Figure 2*A* shows examples of four types of trials. A GP trial began with a fixation displayed for 500 ms, followed by a general preparation stage with a duration randomly ranging from 1500 to 2000 ms with an increment of 100 ms. The general preparation stage was cued by two yellow symbols (#%) presented in the center of the screen. Participants prepared to produce sounds, but the symbols did not provide any information about what sound to produce. During the last 400 ms of the general preparation stage, one of the three auditory stimuli (auditory syllable, cough, or pure tone) was presented. After the general preparation stage and a blank period (randomized in a range from 600 to 800 ms), participants were asked to articulate a sound as quickly and accurately as possible according to a visual cue in green that appeared in the center of the screen. Three visual cues, each composed of two green symbols, indicated the sound to produce—visual characters of “ba” for speaking the syllable/ba/, “<∼” for producing cough sound, and “–” cued participants to hum the first lexical tone (flat tone) in Mandarin Chinese. The reaction time (RT) of the articulation in each trial was recorded as the time interval between the onset of the green visual cue and the onset of participants’ vocal responses.

GP_NS_ trials were similar to GP trials, except that no sound was presented in the last 400 ms of the general preparation stage. The GP_NS_ trials were included in the experiment to ensure that preparation in the general preparation stage was independent of auditory probes in GP trials. That is, the preparation should occur after the preparatory visual cue and be available during the presentation of the auditory probe in GP trials. In the NP trials, participants were asked to perform the articulation tasks without any preparation. By comparing RTs in NP trials with those in the GP trials or GP_NS_ trials, we could quantify general preparation in the GP trials or GP_NS_ trials behaviorally. Similarly, comparing RTs in the GP_NS_ trials with those in the GP trials could infer whether general preparation occurred independently of the auditory probe.

The PL trials were marked by two blue symbols “**”. Similar to the general preparation stage in the GP trials, the visual cue was also displayed in a duration randomly selected from 1500 to 2000 ms in an increment of 100 ms. An auditory probe was presented during the last 400 ms of visual cue presentation. The auditory probes played in the PL trials were the same as those in the GP trials. However, In the PL trials, participants listened to the auditory probes passively without any preparation or articulation task. By comparing EEG responses to the auditory probes in the PL trials with those in the GP trials, we could examine whether CD generated in the general preparation stage modulates early auditory responses to the auditory probes.

In summary, a within-subject design with four types of trials (GP, GP_NS_, NP, and PL) was used in this study. Three auditory probes (syllable, cough, and pure tone) were in the trials of GP and PL, yielding six conditions in EEG responses. The experiment consisted of six blocks. Each block included 96 trials, with 24 trials for each type of trial. The number for each of the auditory probes was equal and yielded 48 trials separately for the stimulus of syllable, cough, and tone in GP and PL. The order of trials was randomized. A short break of 1–2 min was provided between blocks.

### Behavioral data analysis

To evaluate the effect of general preparation behaviorally, articulation RTs of the articulation task, the time interval between the onset of the green visual cue and the onset of the vocalization, were compared across different conditions using one-way repeated-measures ANOVA to assess the differences among three conditions (GP, GP_NS_, and NP). *Post hoc t* tests with Bonferroni’s correction were conducted for pairwise comparison between conditions using the Pingouin toolbox (Vallat, 2018).

### EEG data acquisition and preprocessing

EEG signals were recorded using a 32-channel Brain Products actiCHamp recording system. The 32 electrodes over the scalp were placed based on the 10/20 international electrode system. To monitor ocular activity, the EOG was recorded from two additional electrodes, one placed 1 cm lateral to the lateral canthus of the left eye, and the other below the right eye. The electrode impedances were kept under 10 kΩ. The electrode of Cz was used as the online reference. An online low-pass filter with a cutoff at 200 Hz and a notch filter at 50 Hz were used. The EEG data were digitized with a sampling frequency of 1000 Hz.

EEG data preprocessing was performed using MNE-Python (Gramfort et al., 2014). The continuous EEG data were bandpass filtered (0.1–30 Hz). Bad channels were identified visually and repaired using spherical spline interpolation (Perrin et al., 1989). Epochs spanning from −100 to 300 ms related to the onset of the auditory probe were extracted in each trial of GP and PL. Baseline correction was applied using the 100 ms prestimulus period. Epochs with maximum peak-to-peak amplitude exceeding 100 μV on any channel were rejected. Epochs contaminated by eyeblink and movement artifacts were rejected manually. The average rejection rate was 20.85%. The EEG data were rereferenced to the average of all electrodes over the scalp.

### Temporal domain analysis

Event-related potentials (ERPs) were calculated by averaging epochs for each auditory probe and each participant, as well as for three auditory probes combined, yielding four ERP responses (syllable, cough, tone, and three-sound combined) separately in the PL and GP conditions. The global field power (GFP), calculated as the SD of the ERP responses across all electrodes (Lehmann and Skrandies, 1980), was derived using the EasyEEG toolbox (Yang et al., 2018). The GFP responses reflect an overall power change in all electrodes across time, which avoid potential subjective bias in selecting electrodes during analysis. Individual N1 and P2 amplitudes were obtained by averaging the 20 ms responses centered at the peak latency of each component in the GFP waveforms using the TTT toolbox (Wang et al., 2019).

First, to demonstrate the overall inhibitory effects of corollary discharge in general preparation, we conducted a paired *t* test between the GP and PL conditions in the three-sound combined GFP responses, separately for N1 and P2 components. Next, to investigate the modulation effects on each type of auditory probe, an additional three paired *t* tests were performed, each on the syllable, cough, and tone GFP responses, separately for N1 and P2 components. To better connect with the literature and provide more intuitive results, we also performed the ERP analyses based on the most common representative channel of ERP auditory responses: Cz.

### Spatiotemporal analysis

Because the GFP measure is an omnibus index across all electrodes, its statistical power could be limited by noise or lack of signals in any subset of channels. Furthermore, GFP analysis only provides temporal information about the modulation effects. To increase statistical power as well as to further investigate spatial aspects of the modulation effects, the nonparametric spatiotemporal cluster-based permutation test (Maris and Oostenveld, 2007) was performed using the MNE-Python toolbox. For each type of sound, the empirical *t* statistics were first obtained via two-tailed paired *t* tests on the ERP responses between GP and PL conditions at each time point between −100 and 300 ms time locked to the sound onset and in each electrode. Time points in each electrode with absolute *t* values exceeding the threshold (α = 0.05) were identified. Selected time points in all electrodes with *t* values of the same sign (positive or negative) were clustered based on spatiotemporal adjacency. The cluster with maximum points was selected separately for the positive and negative sign *t* values, and the empirical statistics were obtained by calculating the sum of the *t* values within a cluster. The same clustering process was repeated 10 000 times after each time shuffling the condition labels. A null distribution was obtained, separately for the positive and negative sign clusters. The *p*-value of each cluster was determined as the proportion of *t* values in the null distribution that were larger than the empirical statistics.

### Time–frequency analysis

To investigate whether the inhibitory function of corollary discharge modulates the amplitude or the timing of perceptual responses, time–frequency analyses were conducted separately on the aspects of power and phase in several frequency bands. Specifically, longer epochs (−2000 to 2000 ms time locked to auditory probe onset) for each sound in GP and PL conditions were extracted to avoid edge artifacts. Morlet wavelet transform was applied on each of the longer epochs using the function of “tfr_morlet” in the MNE-Python toolbox with the parameter of n_cycles setting to two cycles for each frequency in 1–3 Hz and frequency/2 for other frequencies (4–28 Hz). Power and phase in each frequency at each time point in each electrode were obtained for every condition. Data between −100 and 300 ms were used for further analysis.

For power analysis, the averaged power between −100 and 0 ms was used as the baseline. Power values were normalized by dividing the mean of the baseline and were converted into a log scale. For phase analysis, intertrial phase coherence (ITC) was calculated based on the following equation (Tallon-Baudry et al., 1996; Luo and Poeppel, 2007):

$$\text{ITC}(t,f)={\left(\frac{\sum_{j=1}^{N}\cos\theta_{j}(t,f)}{N}\right)}^{2}+{\left(\frac{\sum_{j=1}^{N}\sin\theta_{j}(t,f)}{N}\right)}^{2}.$$

Power and ITC values were further averaged within the following six frequency bands: the delta (1–3 Hz), theta (4– 8 Hz), alpha (9–12 Hz), low-beta (13–16 Hz), mid-beta (17–20 Hz), and high-beta (21–28 Hz) bands. The nonparametric spatiotemporal cluster-based permutation test was used to assess the significant difference between GP and PL conditions for each sound, separately for power and ITC in each frequency band.

The data and codes in the present study are publicly available on the OSF (https://osf.io/au43q/).

## Results

### Behavioral results

Participants were asked to produce a sound with or without preceding general preparation. A repeated-measures one-way ANOVA on RTs showed a significant main effect of preparation (*F*_(2,38)_ = 37.45, *p *<* *0.0001, partial η^2^ = 0.664). Bonferroni-corrected paired *t* tests revealed that RTs were faster when participants performed the articulation task in GP than in NP (*t*_(19)_ = 6.060, *p* <0.0001, *d *=* *0.875). Moreover, RTs in GP_NS_ was also faster than NP (*t*_(19)_ = 6.970, *p *<* *0.0001, *d *=* *0.947). However, no significant difference was observed between GP and GP_NS_ (*t*_(19)_ = 0.396, *p* = 1, *d *=* *0.028). These results (Fig. 2*B*) replicated the observations in the study by Li et al. (2020) and indicated that participants engaged in general preparation regardless of the existence of an auditory probe, which suggested that CD was available before sound onset and throughout the general preparation stage.

### ERP components results based on all channels revealed overall P2 suppression

ERP responses to all auditory probes combined, including GFP waveforms and topographies of N1 and P2, are shown in Figure 2*C*. Paired *t* tests revealed that no significant difference between N1 amplitude in GP and PL conditions (*t*_(19)_ = 0.517, *p *=* *0.611, *d *=* *0.051), whereas the P2 amplitude in GP was significantly suppressed compared with PL (*t*_(19)_ = 2.528, *p *=* *0.020, *d *=* *0.416). These results supported the hypothesis that CD during motor intention exerted an inhibitory function on auditory neural responses.

To further test the hypothesis of whether CD has a generic inhibitory function and suppresses all sounds that link to articulatory features, even without specific articulatory encoding during the motor intention stage, we examined the modulation effects of CD on each type of auditory probe. Paired *t* tests on the GFP response amplitude revealed a similar suppression in P2 component in responses to cough (*t*_(19)_ = 2.950, *p *=* *0.008, *d *=* *0.517), but not in N1 component (*t*_(19)_ = 1.147, *p *=* *0.266, *d *=* *0.181). However, the suppression effects were absent in responses to the auditory stimuli of the syllable and tone. These null results could be because of relatively weak suppression effects in the responses to different types of sounds and GFP that summarized over all electrodes cannot provide enough statistical power to detect these weak effects. To be comparable with previous studies and offer more straightforward results, we examined the modulation effects based on the most common representative channel of auditory ERP responses: Cz.

### Results of ERP analysis based on the channel of Cz revealed P2 suppression in each type of sound

**Figure 3.**
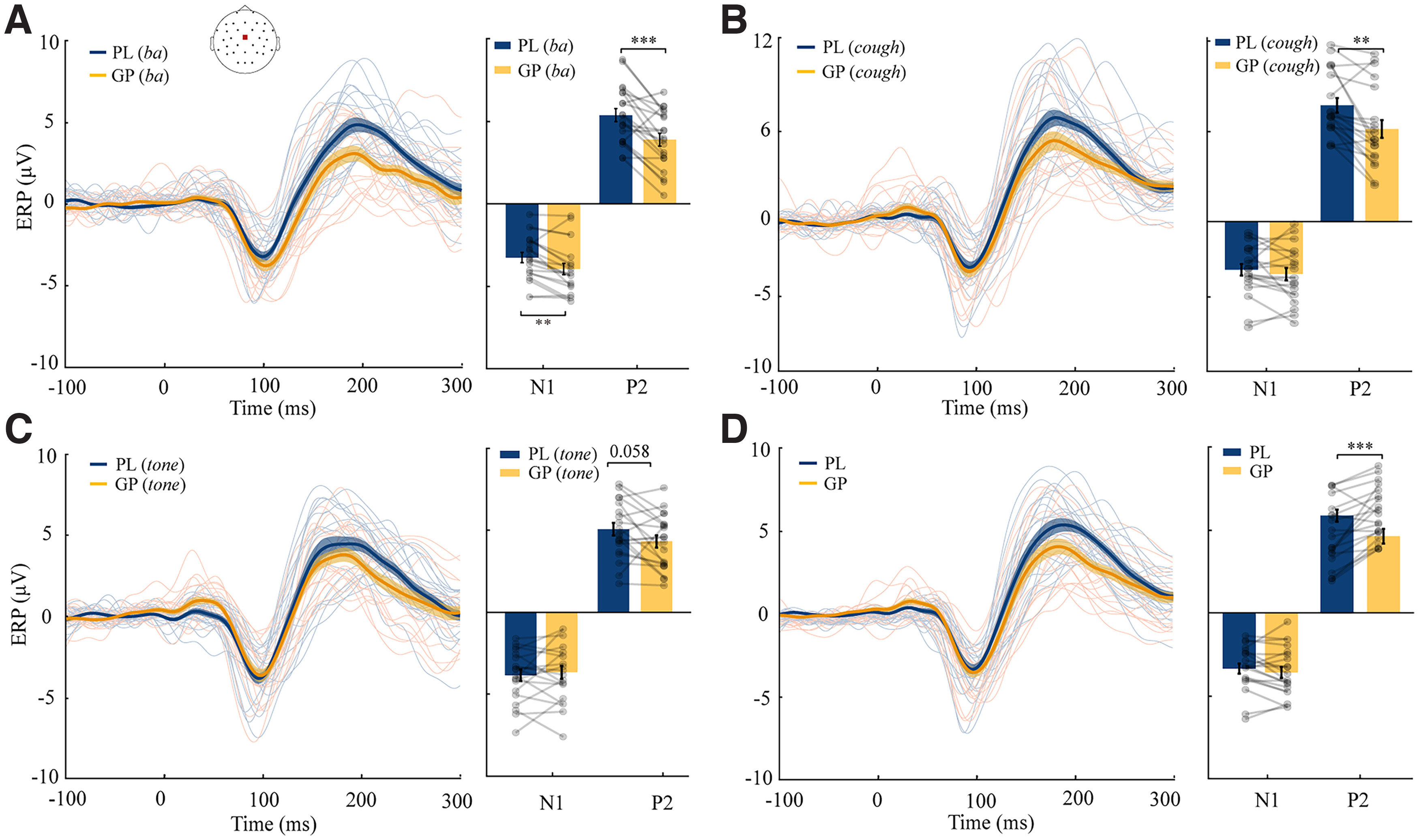
Grand average ERP responses to auditory probes in the representative channel of Cz. The waveform responses are in the left column, and the N1/P2 component responses are in the right column in each panel. ***A–D***, Responses to syllable (***A***), cough (***B***), tone (***C***), and the average across three types of sounds (***D***), respectively. Individual data are superimposed on each plot. Yellow and blue represent GP and PL conditions, respectively. Error bars indicate ±SEM. **p < 0.01, ***p < 0.001.

The results of the representative channel Cz are shown in Figure 3. For ERP responses to the auditory probe of syllable (Fig. 3*A*), paired *t* tests revealed that the amplitude of P2 response in GP was reduced relative to that in PL (*t*_(19)_ = 4.533, *p *=* *0.0002, *d *=* *0.847). For ERP responses to cough sound (Fig. 3*B*), the amplitude of P2 response in GP was less than that in PL (*t*_(19)_ = 3.831, *p *=* *0.0011, *d *=* *0.653). For ERP responses to the pure tone (Fig. 3*C*), P2 suppression in GP only survived a one-tailed paired *t* test rather than a two-tailed test (*t*_(19)_ = 2.017, *p *=* *0.0580, *d *=* *0.437). Additionally, the amplitude of early N1 response in GP was enhanced relative to that in PL for syllable (*t*_(19)_ = 3.872, *p *=* *0.0010, *d *=* *0.473). For ERP responses to all sounds average (Fig. 3*D*), the amplitude of P2 response in GP was suppressed relative to that in PL (*t*_(19)_ = 4.301, *p *=* *0.0004, *d *=* *0.689), consistent with the GFP results. The representative channel analysis revealed inhibition for all types of sounds. To further test the spatial distribution of the effects, we conducted a spatiotemporal cluster analysis by considering the spatial information in addition to the temporal information to further investigate the hypothesis of the generic inhibitory function of CD.

### Results of spatiotemporal cluster-based permutation tests

**Figure 4.**
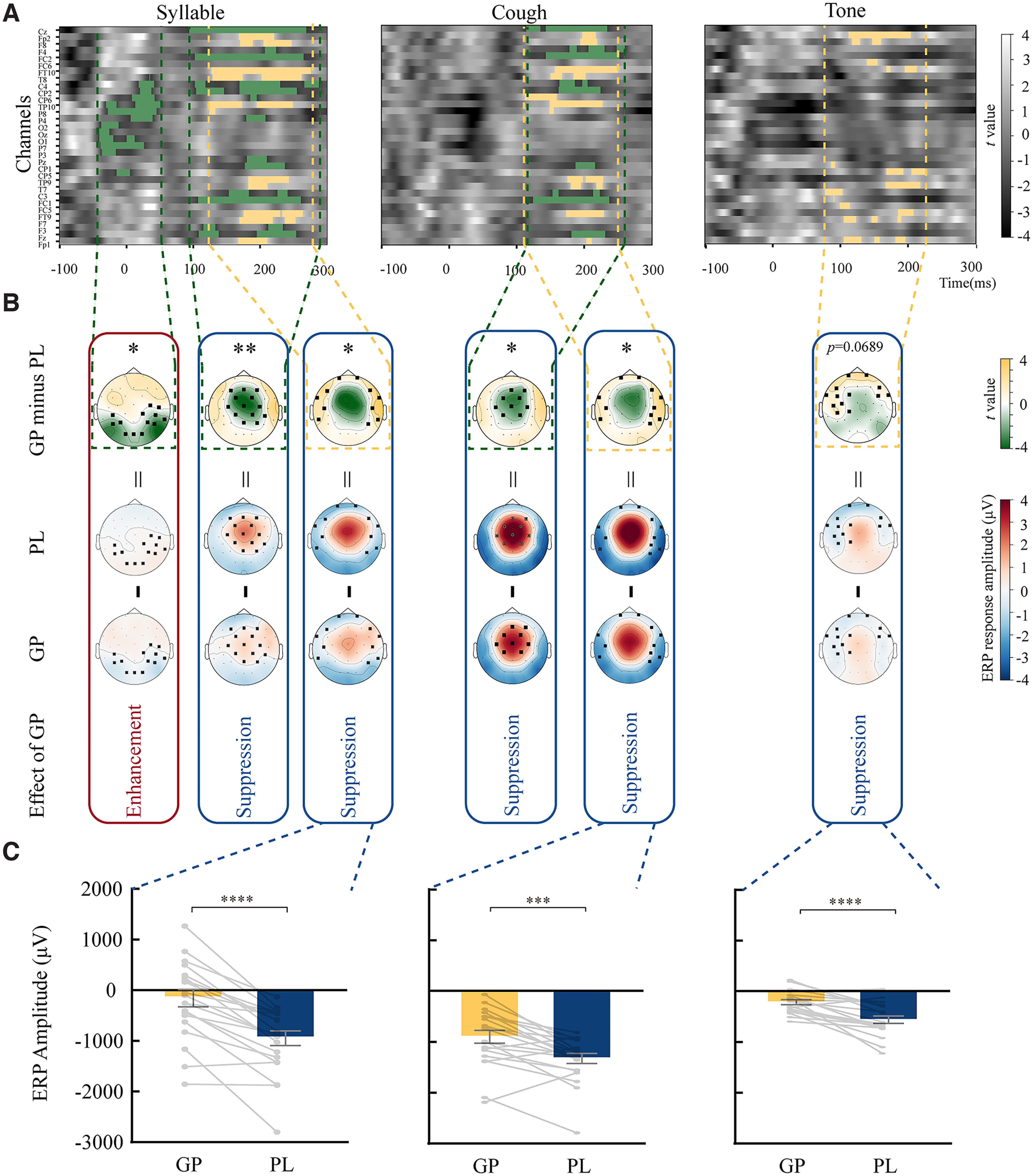
The results of spatiotemporal analysis on ERP responses. Each column indicates the results for syllable, cough, and tone, respectively. ***A***, The results of the spatiotemporal analysis. The *x*-axis represents time relative to the auditory probe onset at 0 ms, and the *y*-axis represents each of the 32 electrodes. The grayscale in the background represents *t* values comparing the ERP responses between GP and PL conditions (GP minus PL). Yellow and green indicate significant clusters with positive and negative *t* values, respectively. ***B***, Topographic representation of the significant spatiotemporal clusters in ***A*** and the raw ERP topographies that derived the significant cluster results. Each topography in the first row represents averaged *t* values across the time interval of each significant cluster in ***A***, indicated by corresponding color dashed lines. The black squares on the topographies indicate the significant electrodes in the cluster. The second and third rows are the topographies of averaged ERP responses across the corresponding time interval of the cluster in the PL and GP, respectively. The black squares on the ERP topographies label the same electrodes in the corresponding significant cluster above. Considering the polarity of ERP responses, the clusters observed in typical latency of auditory responses (100–200 ms after stimuli onset) showed inhibition effects of general preparation on all types of auditory probes—the ERP amplitudes in GP were smaller than those in PL (less positive or less negative in electrodes of positive or negative ERP responses, respectively). ***C***, The summarized results of three ERP clusters with individual data extracted using the group-level clusters as a spatial-temporal filter. To better compare the suppression effect of GP in each sound, the sum of ERP data in a similar cluster of frontal-temporal distribution was presented for each sound. Error bars indicate ±SEM. *p <* *0.05, **p <* *0.01, ***p <* *0.001, ****p <* *0.0001.

To collaboratively reveal the modulation effects in the aspects of spatial distributions and temporal characteristics, we conducted spatiotemporal cluster-based permutation tests. The results of spatiotemporal cluster analysis are shown in Figure 4, separately for each type of sound. For syllable, three significant clusters were found. The first significant cluster (*p *=* *0.0497) appeared around time 0 ms (range, −40 to 51 ms; Fig. 4*A*, statistical parametric heatmap). The spatial distribution of this cluster was mostly over parietal regions, as shown in the topography of the statistical map in the first row of Figure 4*B*. The nature of the modulation effects was further illustrated by examining the raw ERP topographies (averaged amplitudes across the time interval of the cluster) of PL and GP conditions (Fig. 4*B*, last two rows). Responses in the PL condition were ∼0, which presumably reflected random processes during a passive task before auditory probe onset; whereas responses in the GP condition were more negative in the posterior electrodes, which were consistent with neural sources that mediated motor intention and preparatory processes (Desmurget et al., 2009; Tian and Poeppel, 2010). The more negative ERP in GP, compared with random activation in PL, resulted in a negative sign of statistics, which reflected the enhancement effects of general preparation (more absolute magnitude of activation but in electrodes of negative polarity) in a significant cluster of electrodes over parietal regions before auditory probe onset.

The other two significant clusters observed in responses to syllable were both ∼200 ms after the auditory probe onset (Fig. 4*A*). The cluster that had a central spatial layout had negative statistics (*p *=* *0.0038), whereas the one with a peripheral distribution in electrodes over frontal-temporal regions had positive statistics (*p *=* *0.0149; Fig. 4*B*). The adjacent distributions of these two clusters resemble the different polarities in the dipole patterns of ERP topographic responses to the auditory syllable (Fig. 4*B*, last two rows), collaboratively depicting the suppression effects of GP on the neural responses of speech sound. Specifically, the cluster with negative statistics distributed over the central electrodes showed positive ERP values in GP and PL conditions. Responses in GP were less positive than in PL. The comparison between GP with PL hence yielded a significant cluster with negative statistics in this central cluster, reflecting the suppression effects of GP on responses to the auditory probe. Similarly, the cluster with positive statistics was caused by less negative ERP in GP than PL in the peripheral frontal-temporal electrodes, reflecting the inhibition of CD on the response magnitude of ERPs to an auditory syllable. That is, the observed two clusters reflect a significantly smaller magnitude of responses to the auditory syllable in GP than in PL, supporting the suppression effects of CD during GP on the neural responses of speech sound.

For cough, two significant clusters were observed at ∼200 ms (Fig. 4*A*, second column). Similar to those in the syllable, one was located in central regions (*p *=* *0.0274) and the other was in peripheral frontal-temporal regions (*p *=* *0.0485; Fig. 4*B*). These two clusters both reflected the absolute amplitude decrement in responses to auditory probe in GP compared with those in PL, separately for two sets of electrodes that had ERP responses in opposite polarities in the P2 component (Fig. 4*B*, last two rows). Specifically, the central cluster with negative statistics was caused by less positive ERP responses in GP (mean* *=* *2.798 μV) than in PL (mean* *=* *3.912 μV); whereas the peripheral frontal-temporal cluster with positive statistics was caused by less negative ERP responses in GP (mean = −1.571 μV) than in PL (mean = −2.311 μV). These results suggested that CD in GP also induced suppression effects on the responses to nonspeech cough sounds.

For tone, only one cluster was found (from 78 to 225 ms) in peripheral electrodes of frontal-temporal regions (Fig. 4*A*, third column). This cluster had positive statistics that were caused by less negative ERP responses in GP (mean = −0.525 μV) than in PL (mean = −1.359 μV), similar to the one in syllable and cough (Fig. 4*B*). However, this cluster only survived a one-tailed spatiotemporal cluster permutation test but not a two-tailed test (*p *=* *0.0689). These results suggested that CD exerted a weak inhibitory effect on the nonhuman sound of pure tone. Altogether, the results of spatiotemporal cluster analysis suggested that CD suppressed the neural responses to all types of auditory probes. The strength of CD was stronger for speech and nonspeech sounds than for nonhuman sound, which suggested that the strength of the inhibition effects was constrained by the established motor–sensory associations—the generic inhibitory function of CD operates in the pathways that link to the auditory features of human sounds; the CD may not suppress the neural responses to pure tones or may suppress in a gradient manner based on the distance of pure tones from the range of human voice pitch.

To provide direct visualization of individual-level data and comparisons among sounds, we extracted each participant’s data using the group-level clusters as a spatial-temporal filter. For each sound, the results of the cluster with consistent suppression patterns across sounds were presented in Figure 4*C*. For the sum of ERP responses in the suppression cluster of syllable sound, paired *t* tests revealed that the amplitude of P2 response in GP was reduced relative to that in PL (*t*_(19)_ = 7.269, *p *<* *0.0001, *d *=* *1.114). For the sum of ERP responses to cough sound, the amplitude of P2 response in GP was less than that in PL (*t*_(19)_ = 4.437, *p *=* *0.0002, *d *=* *0.835). For the sum of ERP responses to the pure tone, P2 response in GP was significantly suppressed compared with that in PL (*t*_(19)_ = 5.240, *p *<* *0.0001, *d *=* *1.234). These results indicated that the suppression effect of CD was found in each kind of sound, consistent with the results of the spatiotemporal cluster permutation test. To compare the suppression effect of GP across auditory probes, a repeated-measures one-way ANOVA was performed on the differences between the sum of ERP data in the cluster of PL and GP across three types of auditory probes. The results showed a significant effect of sound (*F*_(2,38)_ = 7.980, *p *=* *0.001, partial η^2^ = 0.296). Bonferroni-corrected paired *t* tests revealed that the suppression effect in syllable sound was larger than that in cough sound and tone (syllable vs cough: *t*_(19)_ = 3.419, *p *=* *0.009, *d *=* *0.804; syllable vs tone: *t*_(19)_ = 3.461, *p *=* *0.008, *d *=* *1.131). These results were consistent with the ERP cluster results as well as the component analysis results (Fig. 4) that showed smaller inhibitory effects in tones compared with the other two types of sound.

### Results of time–frequency analysis

To further investigate how CD influenced auditory processes, whether it suppressed the response magnitude or disrupted the timing, we conducted time–frequency analyses using spatiotemporal cluster-based permutation tests, separately for response power and phase. Because the three sounds included in this study had different modulation rates (the cough sound had sharper acoustic onset and hence had relative more energy in the theta band compared with syllable and tone sounds), we first conducted the time–frequency analysis to explore the modulation effects in separate delta (1–3 Hz) and theta (4–8 Hz) bands. Next, for a fair comparison with more statistical power, we pooled the two frequency bands together and performed the time–frequency analysis in one lower-frequency band (1–8 Hz) that included the most speech processes for all types of sounds (Giraud and Poeppel, 2012). Because similar results were obtained in separate and combined frequency bands, we elaborated on the results of one lower-frequency band.

**Figure 5.**
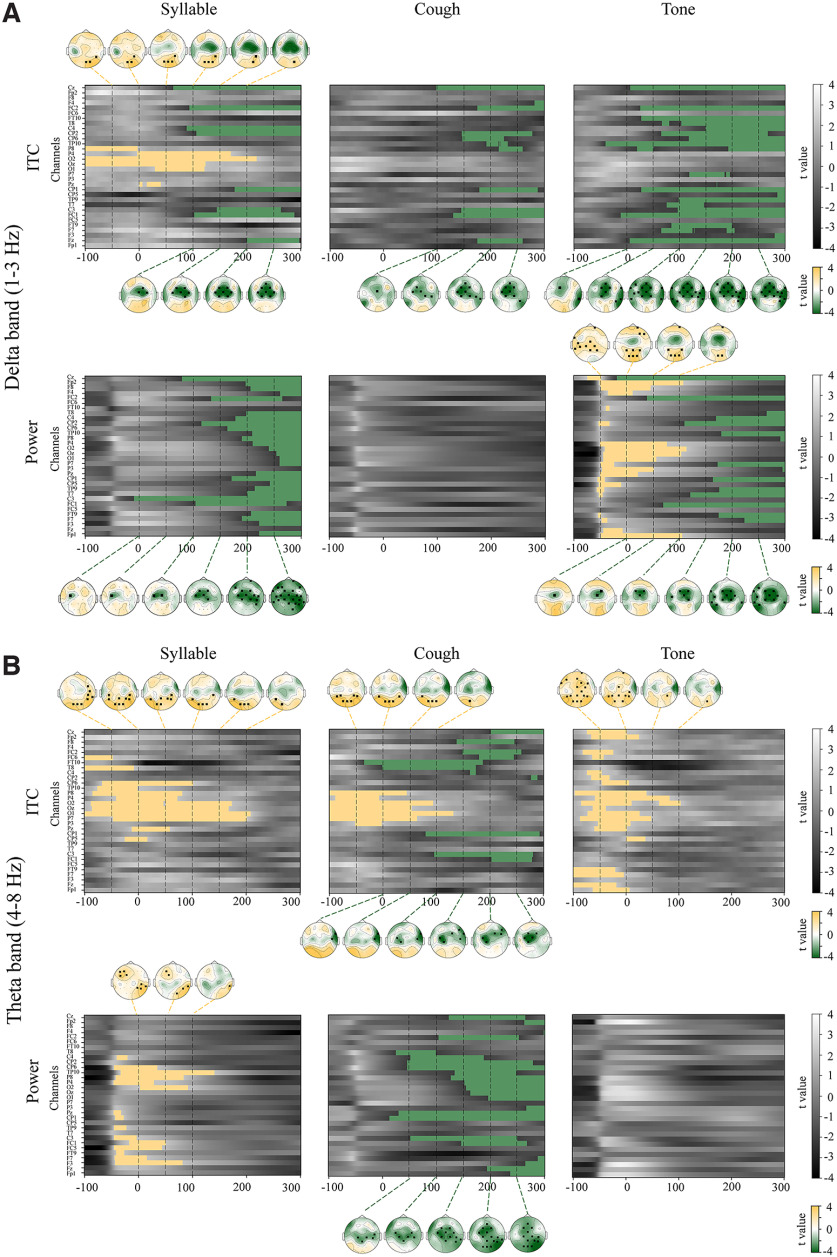
Results of spatiotemporal cluster analysis in the delta frequency band (1–3 Hz) and the theta frequency band (4–8 Hz) for each type of auditory probe, separately for ITC and power. Each column indicates the results for syllable, cough, and tone, respectively. The grayscale images represent *t* values in each of the 32 electrodes across time, obtained by comparing the ITC or power between GP and PL conditions (GP minus PL). The yellow and green indicate clusters with positive and negative *t* values and hence enhancement and suppression effects, respectively. Topographies of averaged *t* values are plotted every 50 ms from −100 to 300 ms when the significant clusters were observed. Significant electrodes in each cluster are marked with black squares on each topography. ***A***, Results in the delta frequency band. The ITC results are presented in the top row. For syllable, two significant clusters were found. Topographies of the first clusters in yellow, spanning from −100 to 200 ms, are shown at the top of the spatiotemporal plots. Significant electrodes in this cluster were mostly located in parietal regions, and some were extended to frontal regions. Topographies of the second cluster in green, spanning from 100 to 300 ms, are shown at the bottom of the spatiotemporal plots. Significant electrodes in this cluster were located in central regions. For cough and tone, one significant cluster in green was found, similar to the second cluster in the results of syllable. Power results are presented in the bottom row. For syllable, one significant cluster was found. For tone, two significant clusters were found. No significant cluster was found in the results of cough. ***B***, Results in the theta frequency band. The top row shows ITC results in the theta band. For syllable and tone, one significant cluster in yellow was found. For cough, two significant clusters were found, which is similar to the results of syllable in the delta band. The bottom row shows the power results in the theta band. For syllable, one significant cluster in yellow was found. For cough, one significant cluster in green was found. For tone, no significant cluster was found.

As shown in Figure 5, syllable and pure tone were suppressed in the delta frequency (1–3 Hz) band for both power (for syllable, *p *=* *0.0082; tone, *p *=* *0.0112) and ITC (for syllable, *p *=* *0.0055; tone, *p *=* *0.0003), whereas inhibition to auditory responses to cough sound was mostly in the theta frequency (4–8 Hz) band (for power, *p *=* *0.0041; for ITC, *p *=* *0.0046). Spectrum analyses of the three acoustic stimuli revealed that the modulation spectrum of cough sound had a wider distribution of 1–8 Hz, compared with auditory syllable of 1–5 Hz and a pure tone of 1–3 Hz. The inhibitory effects on different sounds in corresponding frequency bands indicated that the suppression presumably concentrated in the frequency bands that tracked the acoustic signals.

**Figure 6.**
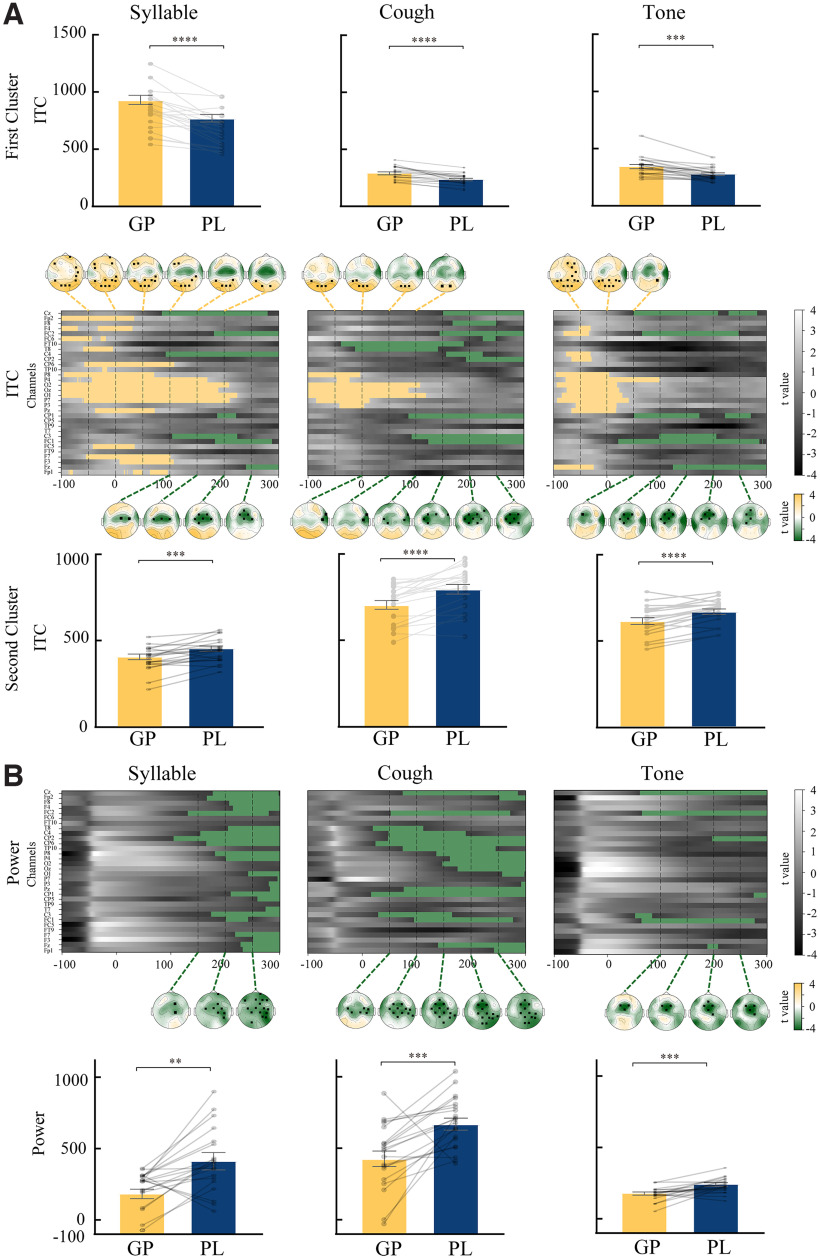
Results of spatiotemporal cluster analysis in one lower-frequency band (1–8 Hz) for each type of auditory probe, separately for ITC and power. Each column indicates the results for syllable, cough, and tone, respectively. The grayscale images represent *t* values in each of the 32 electrodes across time, obtained by comparing the ITC or power between GP and PL conditions (GP minus PL). The yellow and green indicate clusters with positive and negative *t* values and hence enhancement and suppression effects, respectively. Topographies of averaged *t* values are plotted every 50 ms from −100 to 300 ms when the significant clusters were observed. Significant electrodes in each cluster are marked with black squares on each topography. ***A***, ITC results. For each auditory probe, two significant clusters were found. Topographies of the first clusters in yellow, spanning from −100 to 100 ms (for syllable, to 200 ms), are shown at the top of the spatiotemporal plots. Significant electrodes in this cluster were mostly located in parietal regions, and some extended to frontal regions. Topographies of the second cluster in green, spanning from 100 to 300 ms, are shown at the bottom of the spatiotemporal plots. Significant electrodes in this cluster were located in central regions. The summarized results of two ITC clusters of each sound with individual data superimposed are presented at the top and bottom near each cluster separately. ***B***, Power results. For each auditory probe, one significant cluster was found. The clusters were observed from 100 to 300 ms after sound onset in central regions. The summarized results of the power cluster with individual data superimposed are presented at the bottom near each cluster. Error bars indicate ±SEM. **p <* *0.01, ***p <* *0.001, ****p <* *0.0001.

The results of ITC and power in the lower-frequency band (1–8 Hz) exhibited consistent patterns across all types of auditory probes (Fig. 6), similar to the results in the separate frequency bands. Specifically, for ITC results (Fig. 6*A*), two significant clusters that were distinct in spatial and temporal dimensions were found. The first significant cluster (Fig. 6*A*, yellow) had significantly higher ITC values in GP than in PL (for syllable, *p *=* *0.0002; cough, *p *=* *0.0197; tone, *p *=* *0.0249). This cluster in responses to each type of auditory probe occurred at −100 ms (the earliest time included in the analysis) and lasted until 100 ms after stimulus onset (for syllable, 200 ms). Significant electrodes were mostly located in parietal regions, and some extended to frontal regions. The characteristics of this cluster—occurrence before auditory stimuli, posterior spatial distribution, and more consistent phase coherence in GP than in PL—collaboratively suggested that general preparation for actions increased the timing consistency of neural processing across each instance of preparation.

On the contrary, the second significant cluster (Fig. 6*A*, green) had significantly lower ITC values in GP than in PL (for syllable, *p *=* *0.0499; for cough, *p *=* *0.0016; for tone, *p *=* *0.0232). Moreover, this cluster was apparent in the period of 100–300 ms after sound onset and had a central distribution. These temporal and spatial features of this cluster resembled the configuration of the auditory P2 component. The less consistent phase coherence in GP than in PL in a response component to all auditory probes suggested that CD in general preparation decreased the timing consistency of auditory processing.

For the results of power (Fig. 6*B*), only one significant cluster was observed after sound onset (for syllable, *p *= 0.0185; for cough, *p *=* *0.0069; for tone, *p *=* *0.0475). This cluster indicated less power for neural signals in GP than for those in PL. The decrement in power was sparse in tone and more prominent for cough and syllable, consistent with the ERP results. These results suggest that CD during general preparation dampened response power ubiquitously for all auditory stimuli, but the quantity of the power decrease may depend on the established associations between the features in articulation and its auditory consequences. No consistent differences were observed in other frequency bands either for ITC or power. Together, these results suggested that the generic inhibition functions of CD manifested in the modulation of both power and timing of perceptual processes in low-frequency bands. Modulation on the process timing applies equally to each type of auditory probe, whereas modulation on process power may depend on the degree of overlaps between features in articulatory and auditory domains.

Similar to Figure 4*C*, individual data of the sum of power were presented in the last row of Figure 6*B*, and the sum of ITCs in each significant cluster was presented in the top and bottom rows of Figure 6*A* separately. First, all paired *t* tests between GP and PL on each measure were significant (all *p* values* *<* *0.05), consistent with the results of the time–frequency cluster analysis. To compare the suppression effect of GP across auditory probes, repeated-measures one-way ANOVA was performed on the difference between PL and GP, separately for ITC and power. All results showed a significant effect of sound (the first ITC cluster: *F*_(2,38)_ = 11.97, *p *=* *0.0004, partial η^2^ = 0.387; the second ITC cluster: *F*_(2,38)_ = 4.993, *p *=* *0.012, partial η^2^ = 0.208; power: *F*_(2,38)_ = 4.929, *p *=* *0.013, partial η^2^ = 0.206). Bonferroni-corrected paired *t* tests for the first ITC cluster revealed that the enhancement effect in the first ITC cluster in syllable sound was larger than in cough sound and tone (syllable vs cough: *t*_(19)_ = 3.948, *p *=* *0.003, *d *=* *1.057; syllable vs tone: *t*_(19)_ = 3.563, *p *=* *0.006, *d *=* *0.939). For the second ITC cluster, the *post hoc* paired *t* tests revealed that the suppression effect in the second ITC cluster in syllable sound was smaller than in cough sound (*t*_(19)_ = 2.766, *p *=* *0.037, *d *=* *0.702). The paired *t* tests on power revealed that the suppression effect in the power cluster in tone was significantly smaller than in syllable and cough sound (tone vs syllable: *t*_(19)_ = 2.947, *p *=* *0.025, *d *= 0.821; tone vs cough: *t*_(19)_ = 3.089, *p *=* *0.018, *d *=* *0.963). These results suggest that the smaller inhibitory effects on tones compared with the other two types of sound were more consistent in the modulation of power.

We also conducted a spectrotemporal cluster analysis in the middle of the preparation stage (0.5–1.1 s after visual cue onset, a period without possible contamination of visual fixation and subsequent auditory probes). The results showed a similar power decrease in the lower-frequency band in both GP_NS_ and GP conditions compared with the PL condition, suggesting the availability of motor signals in the early stage of motor intention.

## Discussion

We investigated the function of the motor signal generated in the early stage of motor intention. With a delayed articulation paradigm, including three different types of sounds to be produced in the articulation task, we found that the motor signal during motor intention contained no specific information about the sound and suppressed later auditory neural responses to all types of sounds, including speech (syllable/ba/), nonspeech (cough), and nonhuman sound (pure tone). The inhibitory effects were stronger for sounds that humans can produce than for nonhuman sounds. Moreover, we found that the inhibitory modulation of CD was mediated by dampening response amplitude and adding temporal variance to sensory processes. These results suggest a generic inhibitory function of CD that is implemented in the form of modulations on neural response magnitude and timing.

We observed suppression of auditory responses caused by motor signals in the stage of motor intention (Fig. 2). These results are consistent with our previous findings (Li et al., 2020) and suggest that motor signals can transmit to sensory regions in the earliest stage of action. In addition, CD suppressed the neural responses to auditory probes in general preparation, when participants did not know any specific information about actions or consequences of actions. This finding indicates that the inhibitory CD is generated early in the motor intention stage, consistent with the observations that suppression effects were absent when the action is involuntarily triggered without movement intention (Timm et al., 2014). This early onset of motor signals, complementary to commonly observed suppression at the time of action (Blakemore et al., 1998; Ross et al., 2001; Aliu et al., 2009), serves the computational purpose of monitoring throughout the time course of action (Eliades and Wang, 2008; Tian and Poeppel, 2015).

More importantly, the early available CD takes a generic form of inhibition, as the inhibition function modulates all types of sounds (Figs. 3, 4, 6). The generic inhibitory function of CD found in the study was consistent with the previous findings that both speech sounds and nonhuman sounds (pure tone) were suppressed during speech production (Houde et al., 2002). This nonspecific form of prediction may provide the probability of self-induced sensory consequence without the demand for specific representation and hence establish the agency in motor intention (Blakemore and Decety, 2001; Desmurget et al., 2009). Moreover, the observed generic inhibitory function mediates the presupposition of a theoretical mechanism that motor signals increase the signal-to-noise ratio (SNR) of perceptual responses (Reznik and Mukamel, 2019).

Furthermore, we found that the intensity of suppression effects was associated with the distance between feedback sounds and the sounds that humans can produce. Specifically, the strength of inhibition was stronger for the auditory stimuli of syllables and cough than pure tones (Figs. 3, 4, 6). These results are consistent with those of previous studies in which the strength of suppression effects correlated with the action–perception association established via learning—suppression was strongest for the tones with the frequency that paired with action during training, whereas the suppression strength decreased in neurons with auditory receptive fields of adjacent frequencies (Schneider et al., 2018). In the present study, the 500 Hz pure tone was off the normal pitch voice that humans’ vocal folds usually produce. The less suppression of general preparation on the nonhuman sound of pure tone could result from connection strength differences in different associations between motor and auditory areas. The associations between motor and auditory systems for sounds that humans can produce, including speech and nonspeech sounds, are strengthened via everyday pronunciation. Whereas the motor system only links to the auditory features of nonhuman sounds that overlap with features of sounds that humans can produce, fewer or none link to the auditory features that humans cannot produce. Via these available links, the CD transmits and modulates auditory processes, but less strength in the links yields less suppression for nonhuman sounds, even in the generic inhibitory function of CD during general preparation. That is, the motor signal of CD during the intention stage in human articulation does not contain specific information about the sounds that humans can produce, but the CD may still be constrained by the established action–perception associations and has less influence on the auditory processes of nonhuman sounds.

We analyzed EEG signals both in the temporal domain (ERP) and time–frequency domain (power and ITC). Each of these analyses reveals phase-locked and non-phase-locked aspects of EEG data. Specifically, the ERP was obtained by averaging epochs that were time locked to the sound onset. This ERP analysis in the temporal domain amplified the SNR of signals that phase locked to the events; whereas, induced power indicates the response strength of non-phase-locked signals in a certain frequency band, and ITC quantifies phase consistency across trials in the time–frequency domain. The combination of power and ITC yields the effects in ERP. Using these three complementary measures, we found that the generic inhibitory function of CD was implemented both in the modulations of response power and timing. As shown in Figure 6, suppression effects of general preparation were observed both in power and phase coherence for every type of sound probe in the low-frequency band (1∼8 Hz). These results of spectral–temporal analyses are consistent with ERP results (Figs. 3, 4) and demonstrate that the neural modulation mechanisms of CD on sensory processing are dampening response amplitude and increasing temporal variance.

We observed that the inhibition effects manifested in both ITC and power, but with different modulation patterns (Fig. 6). The dissociation between power and phase hints at potential processes of generic inhibition modulation of CD: CD may influence the timing of processing for all sensory features over auditory cortices, then, based on the strength of established connections between motor and sensory features, the detailed inhibition was realized by manipulating the rate of responses and hence the response power. Moreover, “adding noise” could be more “economic” than precisely manipulating neural sensitivity. Increasing temporal variance in the neural phase decreases the probability of alignment between external stimuli and the high-excitability state of the neural phase (Schroeder and Lakatos, 2009; Giraud and Poeppel, 2012). When no content information is available during general preparation, the temporal manipulation on the neural phase primarily mediates the suppression effects over vast neural populations. When motor signals become concrete, especially when action is executed, the modulation on response amplitude dominates the suppression effects precisely on a specific auditory target (Houde et al., 2002; Baess et al., 2011).

We did not find the suppression in the N1 component that was observed in our previous study (Li et al., 2020). The absence of N1 suppression could be because of the different nature of motor signals induced by important experimental differences between the two studies. In the present study, the CD is more general because of the inclusion of three types of sounds. The uncertainty of what types of sound to produce means that even the categorical information of the auditory consequences could not be established during preparation. Whereas in the previous study, the CD contained specific information in a sound category because the subsequent articulation task was only about syllables. Our previous studies suggest that the more concrete and detailed the prediction about the sound from the motor signals, the earlier the modulation effects occurred (Tian and Poeppel, 2013, 2015; Tian et al., 2018). The more abstract “prediction” rather than “specific” prediction of a particular type of sound in the current study may modulate the effects in the later perceptual component because the component of P2 is more relative to abstract categorical coding (Bidelman et al., 2013; Mankel et al., 2020).

The observed N1 enhancement for syllables could be the result of motor intention interacting with speech sounds. We observed the ITC increases caused by motor intention around the onset of the auditory probe and extending to the period that overlapped with N1 latency. Previous studies have demonstrated that the phase at the theta range automatically synchronized with subsequent perceptual responses as early as in the motor planning stage (Tomassini et al., 2017). The observed increased consistency in phase probably reflects the interaction of motor preparation and auditory stimuli, as the motor intention may facilitate the onset of auditory processing, especially for speech sounds. This facilitation could even be as early as in the subcortical pathway, as the studies in vision and eye movement suggest that the CD signals can be available in the colliculus and thalamus (Cavanaugh et al., 2020).

The coexistence of generic inhibitory effects at the latency of P2 and mixed effects at the latency of N1 could be the results of our specific paradigms in the combination of the recording methods used in this study. We designed this study by exploiting the modulation effects of the action on auditory perception. However, the EEG recordings with low spatial resolution could not clearly separate the neural sources of motor preparation and auditory processes, especially at the sound onset. Future studies using methods that have both high temporal and high spatial resolutions, such as intracranial EEG, would offer further evidence distinguishing the sources of CD and its modulation effects in the auditory cortices. Moreover, we used the auditory stimuli with a male voice. Separating participants into two gender groups would provide further evidence investigating the gradient suppression effects based on the distance of the auditory stimuli from the predictive auditory consequences, just like our observations of less suppression for pure tones. However, the random recruitment of participants did not give us enough power to test this interesting point. Future studies can explore the gradient modulation effects in the direction of gender differences.

Using the delayed articulation paradigm, we observed that corollary discharge can be available in motor intention and take a generic form of modulation function to suppress all types of sounds. The generic inhibition function was constrained by the strength of associations between motor and auditory features, and realized by adjusting the amplitude and timing of neural responses. By dissecting the motor-to-sensory transformation signals in functional and temporal dimensions, our results suggest a functional granularity of corollary discharge that mediates the dynamics of motor-to-sensory transformation to fulfill distinct computations in sensorimotor integration and motor control.

## Synthesis

Reviewing Editor: Niko Busch, Westfalische Wilhelms-Universitat Munster Decisions are customarily a result of the Reviewing Editor and the peer reviewers coming together and discussing their recommendations until a consensus is reached. When revisions are invited, a fact-based synthesis statement explaining their decision and outlining what is needed to prepare a revision will be listed below. The following reviewer(s) agreed to reveal their identity: Ana Pinheiro. Note: If this manuscript was transferred from JNeurosci and a decision was made to accept the manuscript without peer review, a brief statement to this effect will instead be what is listed below.

# Synthesis

Both reviews highlight the merit of your study, but they also agree on the need to improve the manuscript further. Specifically, they make several suggestions for discussion some of the inconsistencies in the results and the limitations of the study design, and to tone down a few stements that may not be fully backed up by the results. This includes some of the points that were already addressed in the previous rebuttal letter, but not yet in the manuscript itself.

Finally, I would like to ask you to consider making data and code publicly available.

# Reviewer 1

Thank you for the opportunity to review this interesting paper. I was not a reviewer on the initial submission, but I read through the initial reviews and the careful and thoughtful response from the authors. Overall, I think this paper would make a nice contribution to the literature on sensorimotor processing, pending a few edits/changes, which I outline below.

1. One of the main findings is that inhibition of CD in motor intention is mediated by dampening response amplitude and adding temporal variance to sensory processes. The manuscript would benefit from a discussion of these differential effects. For example, what is the function of increased temporal variance in preparing to speak without knowing what to say (vs. dampening response power)? Relatedly, the hypotheses formulated on p. 5 should be justified with links to existing literature.

2. Another finding that deserves further discussion is the enhanced N1 amplitude in GP relative to PL for syllables (p. 16).

3. I find the discussion of P2 effects not sufficiently convincing. On p. 29, the authors say: “The more abstract ‘prediction’ rather than ‘specific’ prediction of a particular type of sound may make the modulation effects in the current study in the later perceptual component, because the component of P2 is more relative to abstract categorical coding (Bidelman, Moreno, & Alain, 2013; Mankel & Bidelman, 2020).” Could the authors elaborate further?

4. The limitations of the study (some acknowledged in the Response Letter) should be discussed.

Minor issues:

Some sentences should be rephrased for increased clarity. For example:

-P. 4: “That suppression decreased as the frequency of tones deviated from the frequency associated with actions... offers hints supporting this conjecture”.

-P. 19: “The generic inhibitory function of CD operates in the pathways that link to the auditory features that human can produce, and may not suppress or suppress in a gradient manner to pure tones that outside the range of human voice pitch”.

-P. 20: “significant suppressed"

-P. 27: “Each of these analyses reveals certain aspects of EEG data” (specify).

-P. 27: “...can product...”

-P. 28: “The observed increases of consistency in phase probably reflect the interaction of motor preparation and auditory stimuli, as the motor intention may facilitate the onset of auditory processing, even could be as early as in subcortical auditory pathway” (please elaborate further).

-P. 29: “The uncertainty of what types of sound to produce makes even the categorical information could not be established during preparation”.

# Reviewer 2

The authors submitted a revised version of the manuscript with additional analyses and expanded discussion. I thank the authors for their detailed and elaborated response. I still have a few comments on the revised version of the manuscript.

Lines 58-59. The authors write that “The operation of internal forward model has been assumed to rely on the inhibitory modulation of the CD and EC on sensory processing”. I think it is worth mentioning here that motor modulations can also result in enhanced sensory processing. For example, see recent preprint by Press, Thomas and Yon - “Cancelling cancellation? Sensorimotor control, agency, and prediction”.

Line 78. The authors write that “Pure tones partially overlap with...”. I think it will be clearer to phrase it negatively, since the authors want to convey the point that pure tones are not entirely within the human sound repertoire. For example, adding “only partially overlap” makes the point clearer.

Lines 294 and 316 - the subtitles are misleading; I’d explicitly mention that the declared effects relate only to the P2 component (especially given that the Cz N1 analysis showed enhancement).

Line 329 - I am not a native English speaker, but I believe the sentence “analysis revealed the inhibition for all types of sounds” should be without “the”.

Lines 423-442 - Hertz specification should come after “band” - e.g., line 443 - lower-frequency band (1-8 Hz)

Lines 501-503 I believe my previous comment about the misleading statement was not understood by the authors. The authors originally reported no significant GFP P2 modulation for syllable and tone sounds between GP and PL conditions. Following revision, the authors added a separate analysis only of the Cz channel and report more consistent P2 modulation across these two conditions. However, this analysis revealed enhancement of the N1 for syllable. Moreover, like I commented on the previous version of the manuscript, evidence for modulated response to pure tones in the ERP clustering analysis comes from the peripheral channel clusters which can be a simple dipole activity. Other analyses performed by the authors show more consistent patterns (like the ITC and power analyses) that are also more spatially localized around the typical auditory responsive channels. These are mixed results and they do not justify the categoric statement that “we found that the motor signal during motor intention contained no specific information about the sound and suppressed auditory neural responses to all types of sound”. This part should be revised and reflect more carefully and faithfully the results reported in the study. One suggestion is to be more specific about the temporal properties of the suppression or about the signal type in which consistent modulations were observed.

I think replicating the power decrease in the GPns condition is a great addition to the manuscript. If the authors are reluctant to add this analysis as a figure, I would at least mention it in the text.

One thought that came to my mind while I was revising the manuscript is whether the authors expect that female participants will show different modulations in response to male sounds? Your syllable stimuli were male and you had female participants in your cohort. This can be a nice way to test your hypothesis about the specific modulatory nature of motor signals - female participants can produce speech, but not MALE speech. This is not a request for additional analysis, but perhaps an interesting avenue to explore in the future.

## Author Response

Synthesis Statement for Author (Required):

# Synthesis

Both reviews highlight the merit of your study, but they also agree on the need to improve the manuscript further. Specifically, they make several suggestions for discussion some of the inconsistencies in the results and the limitations of the study design, and to tone down a few stements that may not be fully backed up by the results. This includes some of the points that were already addressed in the previous rebuttal letter, but not yet in the manuscript itself.

Finally, I would like to ask you to consider making data and code publicly available.

Dear Dr. Busch,

We thank you and the reviewers for your constructive comments. We are glad to see both reviewers acknowledge the merits of our study as well as the significant improvement in the revisions. In this round of revision, we have provided further clarifications and elaborations on results, as well as provided discussions about limitations and potential future work. Moreover, we have made the data and codes publicly available on the OSF (https://osf.io/au43q/). Below please find our point-to-point responses below each comment.

# Reviewer 1

Thank you for the opportunity to review this interesting paper. I was not a reviewer on the initial submission, but I read through the initial reviews and the careful and thoughtful response from the authors. Overall, I think this paper would make a nice contribution to the literature on sensorimotor processing, pending a few edits/changes, which I outline below.

1. One of the main findings is that inhibition of CD in motor intention is mediated by dampening response amplitude and adding temporal variance to sensory processes. The manuscript would benefit from a discussion of these differential effects. For example, what is the function of increased temporal variance in preparing to speak without knowing what to say (vs. dampening response power)? Relatedly, the hypotheses formulated on p. 5 should be justified with links to existing literature.

Re: Thank you for this excellent suggestion. The hypothesis of increased temporal variance for CD’s inhibition function is derived from the literature on ‘optimal phase of neural oscillations for perception’. According to your suggestion, we have elaborated the hypotheses in the Introduction section by linking them to existing literature.

Moreover, we have added discussions about the differential effects by dampening response amplitude and adding temporal variance in the Discussion section. We copy the updates below.

In the Introduction, ‘Specifically, the effects can be a result of direct gain modulation on response magnitude. Numerous studies have demonstrated that manual actions and speaking dampen the amplitude of neural responses to sounds (Houde et al., 2002; Baess et al., 2011). Whereas in the temporal dimension, it has been suggested that the phase of neural oscillations can be reset and aligned with upcoming external stimuli to boost the sensitivity of neural encoding (Schroeder and Lakatos, 2009; Giraud and Poeppel, 2012; Tomassini et al., 2017; Teng et al., 2020). If CD influences the alignment between neural phase and auditory stimuli, similar suppression effects can be achieved.’

In Discussion, ‘We observed that the inhibition effects manifested in both ITC and power, but with different modulation patterns (Fig. 6). The dissociation between power and phase hints at potential processes of generic inhibition modulation of CD -- CD may influence the timing of processing for all sensory features over auditory cortices; then based on the strength of established connections between motor and sensory features, the detailed inhibition was realized by manipulating the rate of responses and hence the response power. Moreover, ‘adding noise’ could be more ’economic’ than precisely manipulating neural sensitivity. Increasing temporal variance in the neural phase decreases the probability of alignment between external stimuli and the high excitability state of the neural phase (Schroeder and Lakatos, 2009; Giraud and Poeppel, 2012). When no content information is available during general preparation, the temporal manipulation of the neural phase primarily mediates the suppression effects over vast neural populations. When motor signals become concrete, especially when action is executed, the modulation of response amplitude dominates the suppression effects precisely on specific auditory targets (Houde et al., 2002; Baess et al., 2011).’

2. Another finding that deserves further discussion is the enhanced N1 amplitude in GP relative to PL for syllables (p. 16).

Re: Thank you for the suggestion. The enhanced N1 amplitude in GP relative to PL shown in the ERP results for syllables is consistent with strong early enhancement results in the ITC analysis, which could be the result of the interaction between motor preparation and sound. We have added these discussions in the Discussion section and copied them below.

’The observed N1 enhancement for syllables could be the results of motor intention interacting with speech sounds. We observed the ITC increases caused by motor intention around the onset of the auditory probe and extending to the period that overlapped with N1 latency. Previous studies have demonstrated that the phase at the theta range synchronized with subsequent perceptual responses as early as in the motor planning stage (Tomassini et al., 2017). The observed increased consistency in phase probably reflects the interaction of motor preparation and auditory stimuli, as the motor intention may facilitate the onset of auditory processing, especially for speech sounds.

This facilitation could even be as early as in the subcortical auditory pathway, as the studies in vision and eye movement suggest that the CD signals can be available in the colliculus and thalamus (Cavanaugh et al., 2020).’

3. I find the discussion of P2 effects not sufficiently convincing. On p. 29, the authors say: “The more abstract ‘prediction’ rather than ‘specific’ prediction of a particular type of sound may make the modulation effects in the current study in the later perceptual component, because the component of P2 is more relative to abstract categorical coding (Bidelman, Moreno, & Alain, 2013; Mankel & Bidelman, 2020).” Could the authors elaborate further?

Re: Thank you for this comment. Motor prediction about the auditory consequences could be hierarchical. Our previous studies suggest that the more concrete and detailed prediction, the earlier the modulation effects occurred (Tian and Poeppel, 2013, 2015; Tian et al., 2018). In the general preparation stage for articulation, participants need to prepare for articulating three categories of sounds. The motor signal generated in this stage could be predictive at the level of multiple sound categories rather than specific sound types. That is, a more abstract ‘prediction’ is available in the general preparation.

Accordingly, the more abstract ‘prediction’ rather than ‘specific’ prediction of a particular type of sound may make the modulation effects in the current study in the later perceptual component, because the component of P2 is more relative to abstract categorical coding (Bidelman, Moreno, & Alain, 2013; Mankel & Bidelman, 2020). We have updated and clarified in the Discussion.

4. The limitations of the study (some acknowledged in the Response Letter) should be discussed.

Re: We have added the limitations of the study in the Discussion and copied below.

’The coexistence of generic inhibitory effects at the latency of P2 and mixed effects at the latency of N1 could be the results of our specific paradigms in the combination of the recording methods used in this study. We designed this study by exploiting the modulation effects of the action on auditory perception. However, the EEG recordings with low spatial resolution could not clearly separate the neural sources of motor preparation and auditory processes, especially at the sound onset. Future studies using methods that have both high temporal and spatial resolutions, such as intracranial EEG would offer further evidence distinguishing the sources of CD and its modulation effects in the auditory cortices. Moreover, we used the auditory stimuli with a male voice. Separating participants into two gender groups would provide further evidence investigating the gradient suppression effects based on the distance of the auditory stimuli from the predictive auditory consequences, just like our observations of less suppression for pure tones. However, the random recruitment of participants did not give us enough power to test this interesting point. Future studies can explore the gradient modulation effects in the direction of gender differences.’

Minor issues:

Some sentences should be rephrased for increased clarity. For example: -P. 4: “That suppression decreased as the frequency of tones deviated from the frequency associated with actions... offers hints supporting this conjecture”.

Re: We have rephrased the sentence to “A recent study found that the strength of suppression to auditory responses decreased as the frequency of tones deviated from the standard frequency of action consequence (Schneider et al., 2018). This evidence offers hints supporting our conjecture of the gradient suppression effects.”

-P. 19: “The generic inhibitory function of CD operates in the pathways that link to the auditory features that human can produce, and may not suppress or suppress in a gradient manner to pure tones that outside the range of human voice pitch”.

Re: We have updated the sentence to “The generic inhibitory function of CD operates in the pathways that link to the auditory features of human sounds; the CD may not suppress the neural responses to pure tones or suppress in a gradient manner based on the distance of pure tones from the range of human voice pitch"

-P. 20: “significant suppressed”

Re: We have updated accordingly by using the word ‘significantly’.

-P. 27: “Each of these analyses reveals certain aspects of EEG data” (specify).

Re: We have changed the word ‘reveals’ to ‘specifies’.

-P. 27: “...can product...”

Re: We have changed the word ‘product’ to ‘produce’.

-P. 28: “The observed increases of consistency in phase probably reflect the interaction of motor preparation and auditory stimuli, as the motor intention may facilitate the onset of auditory processing, even could be as early as in subcortical auditory pathway” (please elaborate further).

Re: This speculation was derived from an analogy to the recent findings in the vision research. We have elaborated on this point by adding relevant references. The updates are copied below.

‘The observed increases of consistency in phase probably reflect the interaction of motor preparation and auditory stimuli, as the motor intention may facilitate the onset of auditory processing, especially for speech sounds. This facilitation could even be as early as in the subcortical auditory pathway, as the studies in vision and eye movement suggest that the CD signals can be available in the colliculus and thalamus (Cavanaugh et al., 2020).’

-P. 29: “The uncertainty of what types of sound to produce makes even the categorical information could not be established during preparation”.

Re: We have rephrased the sentence to “The uncertainty of what types of sound to produce makes that even the categorical information could not be established during preparation.”

# Reviewer 2

The authors submitted a revised version of the manuscript with additional analyses and expanded discussion. I thank the authors for their detailed and elaborated response. I still have a few comments on the revised version of the manuscript.

Lines 58-59. The authors write that “The operation of internal forward model has been assumed to rely on the inhibitory modulation of the CD and EC on sensory processing”. I think it is worth mentioning here that motor modulations can also result in enhanced sensory processing. For example, see recent preprint by Press, Thomas and Yon - “Cancelling cancellation? Sensorimotor control, agency, and prediction”.

Re: Thank you for the suggestion. We have updated the sentence mentioning enhancement modulation of CD and EC.

"The operation of the internal forward model has been assumed to rely on the inhibitory modulation of the CD and EC on sensory processing (but also see exceptions of enhancement modulation in recent empirical and theoretical studies (Li, Zhu, &Tian, 2020; Press, Thomas, &Yon, 2022))”

Line 78. The authors write that “Pure tones partially overlap with...”. I think it will be clearer to phrase it negatively, since the authors want to convey the point that pure tones are not entirely within the human sound repertoire. For example, adding “only partially overlap” makes the point clearer.

Re: Thanks for the suggestion. We have added the word ‘only’.

Lines 294 and 316 - the subtitles are misleading; I’d explicitly mention that the declared effects relate only to the P2 component (especially given that the Cz N1 analysis showed enhancement).

Re: We have changed the subtitles to “ERP components results based on all channels revealed overall P2 suppression” and “Results of ERP analysis based on the channel of Cz revealed P2 suppression in each type of sound” respectively.

Line 329 - I am not a native English speaker, but I believe the sentence “analysis revealed the inhibition for all types of sounds” should be without “the”.

Re: We have dropped the word.

Lines 423-442 - Hertz specification should come after “band” - e.g., line 443 - lower frequency band (1-8 Hz)

Re: We have changed the sentences.

Lines 501-503 I believe my previous comment about the misleading statement was not understood by the authors. The authors originally reported no significant GFP P2 modulation for syllable and tone sounds between GP and PL conditions. Following revision, the authors added a separate analysis only of the Cz channel and report more consistent P2 modulation across these two conditions. However, this analysis revealed enhancement of the N1 for syllable. Moreover, like I commented on the previous version of the manuscript, evidence for modulated response to pure tones in the ERP clustering analysis comes from the peripheral channel clusters which can be a simple dipole activity. Other analyses performed by the authors show more consistent patterns (like the ITC and power analyses) that are also more spatially localized around the typical auditory responsive channels. These are mixed results and they do not justify the categoric statement that “we found that the motor signal during motor intention contained no specific information about the sound and suppressed auditory neural responses to all types of sound”. This part should be revised and reflect more carefully and faithfully the results reported in the study. One suggestion is to be more specific about the temporal properties of the suppression or about the signal type in which consistent modulations were observed.

Re: Sorry for misunderstanding your previous comments. Thanks for your excellent suggestion about being precise on the temporal properties of consistent modulations.

Therefore, we update this statement by adding precise temporal information about the consistent observed results.

"We found that the motor signal during motor intention contained no specific information about the sound and suppressed later auditory neural responses to all types of sound”.

I think replicating the power decrease in the GPns condition is a great addition to the manuscript. If the authors are reluctant to add this analysis as a figure, I would at least mention it in the text.

Re: Thank you for this suggestion. Since the neural signal during preparation is not our research focus, we added this result to the main text of the manuscript.

"We also carried out a spectrotemporal cluster analysis in the middle of the preparation stage (0.5 - 1.1 s after visual cue onset, a period without possible contamination of visual fixation and subsequent auditory probes). The results showed a similar power decrease in the lower frequency band in both GPns and GP conditions compared to the PL, suggesting the availability of motor signals in the early stage of motor intention.”

One thought that came to my mind while I was revising the manuscript is whether the authors expect that female participants will show different modulations in response to male sounds?

Your syllable stimuli were male and you had female participants in your cohort. This can be a nice way to test your hypothesis about the specific modulatory nature of motor signals - female participants can produce speech, but not MALE speech. This is not a request for additional analysis, but perhaps an interesting avenue to explore in the future.

Re: Thank you for raising this interesting point. This could be another example of a gradient suppression effect based on the distance of the auditory stimuli from the predictive auditory consequences. Unfortunately, we cannot answer this question using the dataset in the present study because of the lack of statistical power after grouping the participants into two groups based on gender. Nevertheless, it is a very interesting point and we have added this in the Discussion and copied below.

’Moreover, we used the auditory stimuli with a male voice. Separating participants into two gender groups would provide further evidence investigating the gradient suppression effects based on the distance of the auditory stimuli from the predictive auditory consequences, just like our observations of less suppression for pure tones.

However, the random recruitment of participants did not give us enough power to test this interesting point. Future studies can explore the gradient modulation effects in the direction of gender differences.’
